# Potential to Reduce Chemical Fertilizer Application in Tea Plantations at Various Spatial Scales

**DOI:** 10.3390/ijerph19095243

**Published:** 2022-04-26

**Authors:** Shaowen Xie, Fen Yang, Hanxiao Feng, Zhenzhen Yu, Xinghu Wei, Chengshuai Liu, Chaoyang Wei

**Affiliations:** 1 School of Environmental and Chemical Engineering, Foshan University, Foshan 528000, China; xiesw@fosu.edu.cn (S.X.); weixinghu1964@163.com (X.W.); 2 Guangdong Laboratory for Lingnan Modern Agriculture, Guangzhou 510642, China; liuchengshuai@vip.gyig.ac.cn; 3 National-Regional Joint Engineering Research Center for Soil Pollution Control and Remediation in South China, Guangdong Key Laboratory of Integrated Agro-Environmental Pollution Control and Management, Institute of Eco-Environmental and Soil Sciences, Guangdong Academy of Sciences, Guangzhou 510650, China; 4 Key Laboratory of Land Surface Pattern and Simulation, Institute of Geographic Sciences and Natural Resources Research, Chinese Academy of Sciences, Beijing 100101, China; yangf@igsnrr.ac.cn (F.Y.); fenghx.17b@igsnrr.ac.cn (H.F.); yuzz.18b@igsnrr.ac.cn (Z.Y.); 5 State Key Laboratory of Environmental Geochemistry, Institute of Geochemistry, Chinese Academy of Sciences, Guiyang 550081, China

**Keywords:** tea plantations, chemical fertilizer, organic fertilizer substitution, reduction potential

## Abstract

Tea is the main commercial crop grown in China, and excessive application of chemical fertilizers in tea plantations is common. However, the potential to reduce chemical fertilizer use in tea plantations is unclear. In this study, Zhejiang Province was selected as the research object to systematically analyze the potential for tea plantation chemical-fertilizer reduction at different spatial scales. The geographic information system-based analytic hierarchy process method and Soil and Water Assessment Tool model were used to determine the chemical fertilizer reduction potential at the province scale and watershed scale, respectively. At the field scale, two consecutive years of field experiments were conducted on a tea plantation. Province-level analysis showed that 51.7% of the area had an average total fertilization intensity greater than 350 kg/hm^2^ and a high reduction potential. Watershed analysis revealed that chemical fertilizer reduction had better potential in reducing total nitrogen and total phosphorus inputs to runoff in the short term, whereas 50% organic fertilizer substitution was the best strategy to achieve long-term effects. The field experiments further proved that organic fertilizer substitution balanced tea growth and environmental protection. This study provides a useful method to investigate strategies to reduce chemical fertilizer use in tea-growing areas.

## 1. Introduction

As a valuable and popular commercial crop worldwide, tea trees (*Camellia sinensis*) are widely planted in the tropical and subtropical areas of China [1,2]. As of 2019, the total tea plantation area of China reached approximately 3.1 million ha, and the yield exceeded 2.7 million tons [3]. The planting area and the output of tea in China are the largest worldwide. To rapidly increase tea yield and quality, two or more times the actual demand for fertilizer are applied; however, an increase in chemical fertilizer use does not necessarily yield a proportionate increase in tea yield with no upper limit [4,5]. A study of the current status of fertilization in tea plantations in China found that 30% of tea plantations overapplied chemical fertilizers [6,7]. The highest rates far exceed the nutrient requirements of tea trees for growth. Excessive input of nutrients from chemical fertilizers can cause serious environmental problems, such as soil acidification, soil compaction, and nitrogen and phosphorus pollution of water bodies [8].

Because of its superior climate and geographical conditions, Zhejiang Province has consistently ranked among the best provinces in China for tea planting area and production. The area and production reached 201.4 thousand hectares and 177,000 tons, respectively, in 2019 [3]. Famous tea products such as Xihu Longjing tea (also known as West Lake Dragon Well tea) and Anji white tea are well known abroad [9,10]. To obtain higher yields and better quality, tea farmers usually apply a large amount of chemical fertilizer to improve the soil nutrients in the tea plantation and promote rapid tea growth. Studies based on surveys of tea farmers have shown that in Zhejiang Province, 30% of tea plantations were planted with nitrogen-fertilizer-application rates of less than 300 kg/hm^2^. Forty-one percent of the tea plantations were planted with more than 450 kg/hm^2^ and only 29% were planted with 300–450 kg/hm^2^ of nitrogen fertilizer [11]. Thus, excessive fertilization generally occurs in tea plantations in Zhejiang Province. In order to effectively improve the soil fertility of tea plantations and reduce the risk of agricultural nitrogen and phosphorus nonpoint-source pollution caused by excessive application of chemical fertilizers, the application of chemical fertilizers in tea plantations must be reduced [12].

To alleviate the problem of excessive fertilizer application in crop planting, the Ministry of Agriculture and Rural Affairs of China formulated the ‘Action Plan for Zero Growth in The Use of Chemical Fertilizers by 2020’ in 2014. The reduction of chemical fertilizer use is gradually gaining more attention and implementation in various provinces of China [13]. Methods such as the direct reduction of chemical fertilizer application and substitution via organic fertilizer application are being used in practical agricultural production [14]. For example, a study found that applying organic–inorganic compound fertilizer with reduced chemical fertilizer in a rice–wheat cropping system may reduce chemical fertilizer use and improve the sustainability of agroecosystems [15]. A similar study conducted in a 10-season continually planted vegetable field revealed that reducing chemical fertilizer use while substituting different amounts of organic fertilizer significantly increased soil organic matter (SOM), catalase activity, and urease activity [16]. Furthermore, spatial heterogeneity among regions causes substantial differences in climate, soil, topography, and other aspects, which should be considered in the formulation of chemical fertilizer reduction strategies [17]. Thus, obvious differences exist among regions in the potential to reduce chemical fertilizer application, and reduction measures corresponding to the region should be adopted. Most importantly, the potential to reduce chemical fertilizer use in tea plantations in Zhejiang Province is unclear. Effective methods to determine this potential at different spatial scales are lacking and require exploration.

Regarding the reduction potential in different regions, methods to analyze the potential for chemical fertilizer reduction in different regions at different spatial scales should be considered. For example, for analysis at the provincial scale, with an area of hundreds of thousands of square kilometers, spatial analysis methods are a good option to summarize possible reductions in different areas. For analysis at the basin scale, with an area of hundreds of square kilometers, simulation can be used to explore the potential of different reduction measures. For small fields of several hundred square meters, field experiments can be used to accurately and quantitatively analyze the environmental effects of various reduction measures. Therefore, considering the current overapplication of chemical fertilizer in tea production areas in Zhejiang Province, this study aimed to explore the potential for chemical fertilizer reduction in tea plantations at different spatial scales. The analysis was performed at three scales: province, watershed, and field. The objectives of this study were as follows: (1) to clarify the potential for chemical fertilizer reduction at the province scale using Zhejiang Province as the research object and geographic information system (GIS)-based analytic hierarchy process (AHP) methods; (2) to simulate the N and P loads under different fertilizer reduction measures and screen potential management reduction measures suitable for tea plantations at the watershed scale using the watershed in the main tea-producing areas of Zhejiang Province as the research object and the Soil and Water Assessment Tool (SWAT) model; (3) to evaluate the potential of environmentally friendly fertilizer reduction measures for tea plantations at the field scale using typical tea plantations in the main tea-producing areas of Zhejiang Province as the research object and performing field experiments under different fertilizer reduction scenarios.

## 2. Materials and Methods

### 2.1. Province-Scale Analysis Method

**Figure 1:**
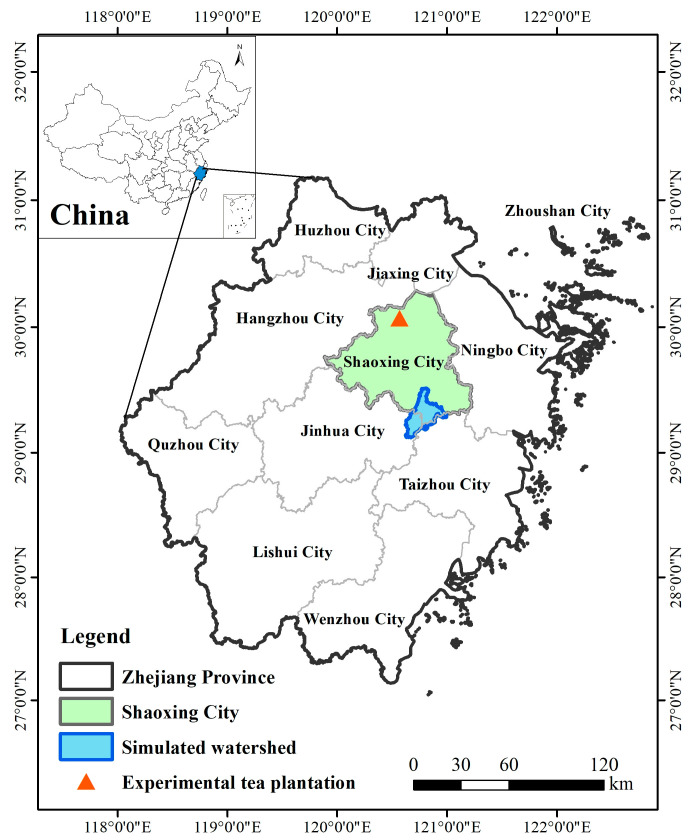
Location of the research site.

Zhejiang Province is located on the southeastern coast of China, and the sufficient heat, abundant precipitation, and suitable light conditions of the region provide a unique natural environment for tea tree growth (Figure 1). This province is a key green tea-producing area in China. In 2018, the area of tea plantations in this province was 199,000 ha, accounting for 6.8% of the country’s total, and the tea output was 184,000 tons, accounting for 7.4% of the country’s total. However, the operating output value of tea plantations in Zhejiang Province was 20.6 billion yuan, accounting for 10.6% of the country’s total. Because of the favorable economic benefits, tea farmers use many chemical fertilizers to maximize tea output [18]. One study investigated the fertilization of tea plantations in 13 cities in Zhejiang Province and found that 41% of tea plantations had excessive fertilization. Among them, the amount of nitrogen fertilizer used in tea plantations in Shaoxing City was the highest, with up to 1566 kg/hm^2^ [11]. Therefore, the use of chemical fertilizers must be reduced in tea plantations in Zhejiang Province.

The growth of tea trees is closely related to the local climate, soil, and topographic environment. These factors directly affect the yield and quality of tea [19]. Additionally, although these factors can be measured using different indicators, the complicated relationships among them cannot be simply expressed by traditional evaluation methods. The integration of multicriteria evaluation methods using geographic information system (GIS) platforms has been a promising area of research [20]. In this study, a GIS-based analytic hierarchy process (AHP) was used to determine the potential for chemical fertilizer reduction in tea plantations throughout Zhejiang Province. In this study, the AHP method was achieved through weight determination, kriging spatial interpolation, and spatial overlay analysis of different indicators, allowing the tea planting potential and chemical fertilizer reduction potential throughout the province to be quantitatively determined. In this study, four climate factors (annual average illumination, annual average precipitation, annual average temperature, and annual average relative humidity), four soil factors (land-use type, soil type, soil texture, and soil erosion degree), and three topographic factors (elevation, slope, and aspect) were chosen for spatial superposition to analyze the suitability of tea planting in Zhejiang Province. These three types of factors were divided into three classes: Class 1, most suitable areas; Class 2, subsuitable areas; Class 3, generally suitable areas. All the factors and factor ratings are shown in Table S2. The meteorological data used in this study were collected from 2008 to 2017 at 65 national meteorological monitoring stations in Zhejiang Province by the China Meteorological Data Network (http://data.cma.cn/, accessed on 12 March 2018). The soil and topographic data used in this study were compiled from the National Earth System Science Data Center (http://www.geodata.cn/, accessed on 12 March 2018).

To analyze the current status of chemical fertilizer application in Zhejiang Province, we used the “Zhejiang Statistical Yearbook” from 2008 to 2017 to obtain detailed statistics on the amount of chemical fertilizer application in all prefecture-level cities in Zhejiang Province. In the past 10 years (2008–2017), the average annual fertilizer application amount in Zhejiang Province reached 899,600 tons, of which the average annual application amount of nitrogen fertilizer was 493,700 tons, the average annual application amount of phosphate fertilizer was 110,800 tons, and the average annual application amount of potassium fertilizer was 7.13 tons. The average annual application amount of compound fertilizer was 223,800 tons (Table S1). In the past 10 years, the application rates of nitrogen, phosphate, and potassium fertilizers in Zhejiang Province have all shown a significant downward trend, but the application rates of compound fertilizers have shown a gradual and slow upward trend, which also reflects the structural changes in the chemical fertilizer application process in Zhejiang Province.

The application intensity of various chemical fertilizers in Zhejiang Province can be calculated from the sown area of crops and the amount of fertilizer application (fertilization intensity = fertilizer application amount/crop sown area). In the past 10 years (2008–2017), the average total fertilization intensity in Zhejiang Province was 344.2 kg/hm^2^. At the same time, in actual production, the amount of fertilization in tea gardens was much higher than that in general grain crops. Therefore, we chose 350 kg/hm^2^ as the baseline value of fertilizer application for subsequent field trials in tea gardens, SWAT model simulation, and provincial reduction assessment. Finally, the suitable areas for chemical fertilizer reduction were classified through the superposition analysis of total chemical fertilizer application. The total area was divided into three classes: most suitable reduction areas (Class 1), where the total chemical fertilizer application intensity is greater than 350 kg/hm^2^; subsuitable reduction areas (Class 2), where the total chemical fertilizer application intensity ranges from 300 to 350 kg/hm^2^; generally suitable reduction areas (Class 3), where the total chemical fertilizer application intensity is less than 300 kg/hm^2^.

### 2.2. Watershed-Scale Analysis Method

The watershed used in the SWAT simulation is located in the southern part of Shaoxing City, Zhejiang Province (Figure 1). It is a typical hilly watershed with the Chengtan River as the main catchment river and a total length of 91 km. Tea plantations are widely distributed in the Chengtan River watershed (accounting for 22.3% of the total area), second only to urban land (accounting for 26.3%), providing a favorable basis to simulate N and P loads and different fertilizer reduction scenarios in the watershed. Additionally, the watershed is closed with only one inlet, effectively avoiding the high uncertainty of simulation results that would arise in the case of multiple inlets; thus, the simulation results of this study are representative. As a distributed hydrological model, the SWAT model can simulate physical processes related to the hydrological cycling of runoff, nutrients, and chemical substances in the watershed [21]. The successful construction of the model database is critical to the effective operation of the model [22]. The database constructed in this study includes digital elevation data (from the Chinese Academy of Sciences (CAS) data cloud), land-use data (from the CAS data cloud), soil-type data (from the State Soil Geographic Database), meteorological data (from the China Meteorological Data Network), and field-measurement-verification data (from inlet monitoring points).

After the database was constructed, the SWAT model was used to divide the watershed into several subwatersheds by determining the smallest watershed area, calculating the parameters of each subwatershed, locating the point source location, and then performing confluence calculations. Finally, data on the discharge and pollution load at the outlet section were obtained. The number of subwatersheds (hydrological response units, HRUs) was determined according to the spatial accuracy of the input data, size of the study watershed, and degree of detail required to achieve the research objectives. The watershed in this study was divided into 9 subwatersheds in total. The distribution of monitoring points, meteorological stations, and land-use types in the studied basin are shown in Figure S1. After the SWAT model is established, the model must be calibrated to confirm that it is suitable for the study area before it can be used for further process simulation. The calibration of the model involves matching the simulation results to the measured data by continuously adjusting the parameters, initial conditions, boundary conditions, and constraints of the model. To verify the accuracy of the SWAT model simulation and evaluate the reliability of the model, the coefficient of determination R^2^ (coefficient of determination) and Nash efficiency coefficient Ens (Nash–Sutcliffe efficiency) between the simulated and measured values of the model are used. In theory, the closer R^2^ is to 1, the higher the degree of agreement between the measured value and the simulated value; the closer Ens is to 1, the higher is the simulation efficiency of the model. Presently, because of factors such as the incomplete collection of spatial data and attribute data, inaccurate estimation of model parameters, and human-induced errors, the simulation results of the models in many studies still show deviations compared with the actual results.

The model was subjected to parameter sensitivity analysis and calibration verification, and continuous adjustments were made through cyclic introduction to finally obtain the best model parameters. SWAT Calibration and Uncertainty Programs (SWAT–CUP) software was used for parameter sensitivity analysis of the model [23]. The coefficient of determination (R^2^) and Nash–Sutcliffe efficiency (Ens) were calculated to evaluate the accuracy of the model simulation results. The runoff, ammonia nitrogen (NH_4_-N), nitrate nitrogen (NO_3_-N), and TP loads were successively calibrated and verified. The findings of Ens > 0.5 and R^2^ > 0.6 verified the acceptability of the results and indicated that the model could be used for subsequent simulation studies [24]. The runoff data for the calibration and verification of the model are all from the “Hydrological Yearbook of the People’s Republic of China—Hydrological Data of the Rivers in Zhejiang, Fujian, and Taiwan”, and the measured water quality data for the model calibration are quoted from previous studies which monitored the river [25].

The calibration and verification results of the model showed that the measured and simulated values of runoff and ammonia nitrogen load were in good agreement during the calibration and verification periods. The measured runoff, ammonia nitrogen load, nitrate nitrogen load, and total phosphorus load, and relative errors of the values and simulated values were within ±20% (Figure S2): R^2^ = 0.64, Ens = 0.57 for the regular runoff rate; R^2^ = 0.61, Ens = 0.52 during the verification period; R^2^ = 0.61, Ens = 0.54 during the regular period of ammonia loading; and R^2^ = 0.52, Ens = 0.54 during the verification period 0.43. Regarding the calibration and verification of the nitrate and total phosphorus loads, the agreement between the measured values and simulated values was also good: Ens = 0.53 during the validation period; R^2^ = 0.56, Ens = 0.44 for nitrate loading; and R^2^ = 0.61, Ens = 0.49 for total phosphorus loading. The calibrated and validated model was used to analyze the spatial distribution characteristics and migration patterns of the N and P loads of each subwatershed under different fertilizer reduction scenarios as well as quantitatively evaluate the environmental effects of different reduced-application scenarios.

### 2.3. Field-Scale Analysis Method

The experimental tea plantation is located in northern Shaoxing City, Zhejiang Province (Figure 1). The field is dominated by a hilly landscape and has been in stable production for more than 30 years. The adult tea trees used for experiments on the plantation were more than 10 years old, and this plantation is one of the largest-scale green tea planting fields in Zhejiang Province. The pilot experimental tea plots were selected within the area of the field mentioned above, in which tea trees have been naturally grown for more than 5 years without manual fertilizer treatments, guaranteeing the reliability of the study in terms of eliminating the influence of external factors. Reductions in chemical fertilizer were performed using organic fertilizer substitution, and the type of organic fertilizer used was rapeseed cake, which is commonly used in tea plantations in Zhejiang Province. Specifically, four treatments were applied, and each was repeated 3 times: 100% chemical fertilizer (0% organic fertilizer substitution; O0); 20% organic fertilizer substitution (O1); 50% organic fertilizer substitution (O2); and 80% organic fertilizer substitution (O3) (Table 1). For all the above treatments, the annual application rate was equivalent to 450 kg/hm^2^ of pure nitrogen, and N: P_2_O_5_: K_2_O was 3: 1: 2. The specific nutrient contents of the fertilizers were as follows: compound fertilizer (N, 150 g/kg; P_2_O_5_, 150 g/kg and K_2_O, 150 g/kg), urea (N, 46%), calcium superphosphate (P_2_O_5_, 12%), potassium sulfate (K_2_O, 60%), and rapeseed cake (N, 52.7 g/kg; P_2_O_5_, 6.9 g/kg and K_2_O, 6.1 g/kg). Many studies have confirmed that reducing fertilizer application directly without considering other corresponding measures will directly affect tea yield. Therefore, reducing fertilizer application by substituting organic fertilizer is a better strategy. The alternative application of organic fertilizer can not only guarantee the yield and quality of tea but also reduce the harm of excessive application of chemical fertilizer. Therefore, the potential of organic manure replacement is essentially the potential of fertilizer reduction [26,27]. Fertilization was performed at the end of March, May, and July, and the proportion of the year with fertilization was 5:3:2, in that order.

All the experimental plots had an area of 21 m^2^, and a 1 m buffer barrier was placed between adjacent plots to prevent water intrusion. Furthermore, according to the flow direction of runoff water, buckets were set at the bottom of each plot to collect runoff water. The effects of fertilization treatments on the SOM, NH_4_-N, NO_3_-N, and TP contents in runoff water were analyzed in late February and October, which represented before and after fertilization, respectively. SOM was measured using an elemental analyzer, runoff water NH_4_-N and NO_3_-N were measured using an automatic chemical analyzer with 1 M KCl soil extractants [28], and TP was determined by inductively coupled plasma–optical emission spectrometry (ICP–OES) [29]. For tea yield determination, the bud density (tea bud number within 0.1 m^2^) during germination and fresh weight yield during harvest in each plot were measured, and tea polyphenols and caffeine from the tea leaves were measured to determine tea quality. The tea yield data were measured on site, and tea quality was determined by the Tea Research Institute, Chinese Academy of Agricultural Sciences.

## 3. Results

### 3.1. Analysis of the Potential for Chemical Fertilizer Reduction in Tea Plantations at the Province Scale

**Figure 2:**
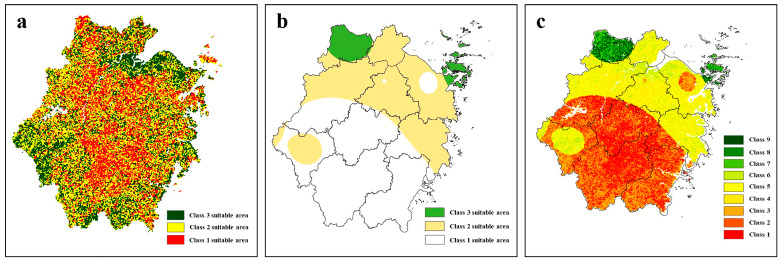
Results of the suitability analysis of chemical fertilizer reduction in tea plantations in Zhejiang Province. Note: (**a**) shows the results of tea planting suitability. Class 1, Class 2, and Class 3 represent the most suitable planting area, subsuitable tea planting area, and generally suitable tea planting area, respectively. (**b**) shows the results of chemical fertilizer reduction suitability. Class 1, Class 2, and Class 3 represent the most suitable reduction areas, subsuitable reduction areas, and generally suitable reduction areas, respectively. (**c**) shows the final results of chemical fertilizer reduction potential in tea plantations of Zhejiang Province. In the suitable planting areas of tea trees of classes 1, 2, and 3, the three grades of chemical fertilizer reduction zones of classes 1, 2, and 3 can be further divided. Finally, 9 corresponding fertilizer reduction areas were formed in the tea planting suitable areas.

The results of tea planting suitability in Zhejiang Province are shown in Figure 2a–c. Specifically, the classification results showed that the subsuitable tea planting area (Class 2) accounted for the highest percentage (55.3%) of the planting area, the most suitable tea planting area (Class 1) accounted for 29.6%, and the generally suitable tea planting area (Class 3) accounted for the lowest percentage (15.1%). Regarding spatial distribution, the most suitable and subsuitable tea planting areas were mainly distributed in the central and northern parts of Zhejiang Province, such as Shaoxing, Hangzhou, and Lishui City. These regions have a favorable combination of precipitation, humidity, and illumination that is very suitable for tea growth. They are also the main tea-producing areas in Zhejiang Province, verifying the accuracy of the suitability analysis [30]. The generally suitable areas for tea planting were mainly distributed in northern Zhejiang Province in the coastal plain and southwestern mountainous areas. These regions fall into this category mainly because of the large annual variations in temperature and precipitation, coupled with low soil fertility, which lead to an unstable tea yield. For suitable tea planting areas, because of their superior conditions, the need for artificial fertilizers is reduced accordingly. However, in the actual tea planting process, compared with the amounts of fertilizer used in other planting areas, equal or even greater amounts of chemical fertilizers are often applied to the generally suitable areas; thus, a higher potential exists to reduce fertilization in the suitable planting areas.

In Zhejiang Province, the average total fertilization intensity reached 344 kg/hm^2^, and considerable variability was observed among different regions in the last 10 years (2008–2017). Regarding spatial distribution, 51.7% of the area was classified as Class 1; this area is crucial to implement fertilizer reduction measures. A total of 41.8% and 6.5% of the area were classified as Class 2 and Class 3, respectively, and these regions were mainly distributed in the northern part of Zhejiang Province. The province has a large area. The potential to reduce fertilization at the provincial scale is closely related not only to the suitability of the tea planting area in the region but also to the actual amount of fertilizer currently invested. A comparison of the spatial distributions of the suitable tea planting area and suitable fertilizer reduction area reveals that these distributions are mostly consistent but that differences exist in some areas. For example, the generally suitable planting area (Class 3) of tea trees usually requires more fertilizer because of the poor environment; thus, this area usually corresponds to the most suitable reduction area (Class 1) or subsuitable reduction area (Class 2). However, for some tea plantations scattered in the steep slope area, although they are located in the most suitable area for tea planting (Class 1), they are also located in the most suitable reduction area for tea planting (Class 1); these tea plantations are the plantations with the highest potential to reduce fertilizer application. Thus, by considering the spatial distribution of the actual amounts of chemical fertilizer application, the potential for chemical fertilizer reduction at the provincial scale can be obtained.

**Figure 3:**
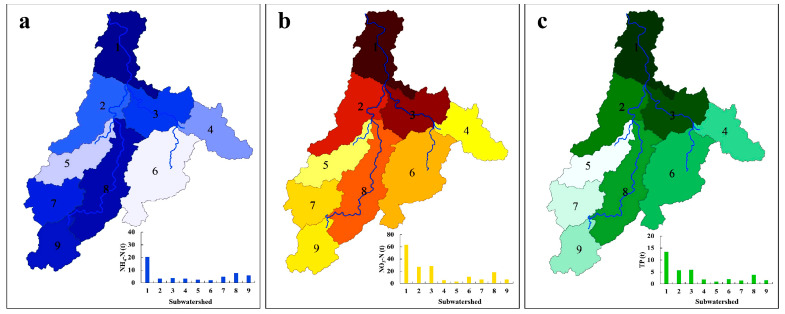
Simulation results of the NH_4_-N, NO_3_-N, and TP loads in tea plantations at the watershed scale based on the SWAT model. Note: (**a**–**c**) show the distribution characteristics of ammonia nitrogen (NH_4_-N), nitrate nitrogen (NO_3_-N), and total phosphorus (TP) in each subwatershed.

After successfully categorizing suitable areas for tea planting and suitable areas for chemical fertilizer reduction in Zhejiang Province, the classes of chemical fertilizer reduction in the differently suitable tea planting areas were calculated. The spatial distribution characteristics of the 9 classes of tea plantations for reduced application of chemical fertilizers are shown in Figure 3c. The detailed rating results and area ratios are shown in Table S3. The subsuitable fertilizer reduction zone in the most suitable tea planting region (Class 2) accounted for the largest percentage in the province, reaching 21.9%, verifying the excessive fertilization occurring in tea plantations in Zhejiang Province. These areas also have the most potential for chemical fertilizer reduction. Theoretically, the more suitable the tea planting area is because of a superior environment, the more the amount of fertilizer can be reduced. However, our analysis revealed that the suitable tea planting area in Zhejiang Province is also the area with the highest amounts of chemical fertilizer application and includes a large available region for application reduction. For example, the subsuitable-fertilizer-reduction area (Class 5) in the subsuitable tea planting area accounted for a high percentage, reaching 16.5%; fertilization must also be reduced here. These areas also have high potential and large regions are available to reduce fertilization.

### 3.2. Simulation of Potential Management Measures to Reduce Fertilization in Tea Plantations at the Watershed Scale

The simulated NH_4_-N, NO_3_-N, and TP loads in the tea plantation watershed based on the SWAT model are shown in Figure 3a–c. The proportional percentage of land usage in each subwatershed was calculated. The total NH_4_-N output of the study basin was 53.6 t, and the ammonia nitrogen output of each subbasin was not the same—sub1 (20.4 t) > sub8 (7.7 t) > sub9 (5.8 t) > sub7 (4.8 t) > sub3 (3.8 t) > sub2 (3.3 t) > sub4 (3.2 t) > sub5 (2.5 t) > sub6 (2.1 t). The total output of NO_3_-N was 169.7 t, much higher than that of NH_4_-N. The output of NO_3_-N was not the same in each subbasin: sub1 (63.3 t) > sub3 (28.4 t) > sub2 (27.2 t) > sub8 (18.4 t) > sub6 (11.1 t) > sub7 (6.5 t) > sub9 (6.4 t) > sub4 (5.3 t) > sub5 (3.3 t). The total output of TP was 36.6 t, which was higher than that of NH_4_-N but lower than that of NO_3_-N. The TP output of each subbasin was not the same: sub1 (13.4 t) > sub3 (6.0 t) > sub2 (5.7 t) > sub8 (3.8 t) > sub6 (2.0 t) > sub4 (1.9 t) > sub9 (1.5 t) > sub7 (1.4 t) > sub5 (0.9 t). In terms of spatial distribution, sub1, sub7, sub8, and sub9 are the sources of the left tributaries of the watershed, and agricultural land dominates. The larger output of NH_4_-N in these subwatersheds may be due to the predominance of agricultural activities in these regions. Regarding NO_3_-N, the total output was 169.7 t, much higher than that of NH_4_-N. The largest output of NO_3_-N was also in sub1 (63.3 t) and 52 times that of the smallest output (sub5). In contrast to the spatial distribution of the NH_4_-N output, the next-highest values of the NO_3_-N output appeared in sub2, sub3, and sub8, which are all adjacent to sub1. This finding was likely due to living organisms being the main source of NO_3_-N in these subwatersheds. Additionally, sub1, sub2, sub3, and sub8 are located in urban centers, and urban residential land accounts for the largest proportion of these subwatersheds. The land-use pattern, dense population, and concentrated sources of industrial emissions may explain the larger output of NO_3_-N in these subwatersheds [31]. The total output of TP was 36.6 t, which was higher than that of NH_4_-N but lower than that of NO_3_-N. The largest TP output was also found in sub1 (13.4 t) and was 37 times that of the smallest TP output (sub5). Similarly, sub1, sub2, sub3, and sub8 are urban centers, where domestic and industrial sources dominate. These phenomena explain why the TP and NO_3_-N outputs showed approximately uniform spatial distributions.

After the SWAT model simulation obtained the N and P inflow loads for each HRU in the whole watershed, the inflow loads of various forms of N and P were calculated by statistical analysis [32]. The proportion of the total nitrogen load of the tea gardens in each subbasin was in the order of sub9 (31.8%) > sub8 (23.9%) > sub6 (19.3%) > sub3 (17.9%) > sub4 (17.7%) > sub7 (16.3%) > sub5 (13.8%) > sub2 (12.2%) > sub1 (6.9%). The proportion of the total nitrogen load in the tea garden was the highest in subbasin 9 and lowest in subbasin 1. Therefore, although the overall total nitrogen load of subbasin 1 and subbasin 2 is exceptionally large, the analysis of the total nitrogen load of specific tea gardens should focus on the variation characteristics of total nitrogen in subbasin 9 and subbasin 8. Tea plantations accounted for 1.9–22.3% in each subbasin, and the average output was 12.1%. The proportion of total phosphorus load in each subwatershed was sub9 (22.3%) > sub6 (18.9%) > sub7 (16.1%) > sub8 (14.2%) > sub4 (11.5%) > sub5 (11.3%) > sub3 (7.9%) > sub2 (4.9%) > sub1 (1.9%). Similar to the TN load, the proportion of TP load in tea gardens was the highest in subbasin 9, and the proportion of TP load in tea gardens was the lowest in subbasin 1, but the proportion of TP load in tea gardens was lower than that of the total nitrogen load in tea gardens, reflecting that the output of phosphorus in tea gardens was less than that of nitrogen. All the details are shown in Figure S4a,b. Because tea plantations account for the second-largest area of the watershed, the average output of tea plantations in each subwatershed reached 17.7%. Although the overall TN loads of sub1 and sub2 were very large, the TN input from tea plantations in sub1 and sub2 was small because the area of tea plantations in sub1 and sub2 was small. Thus, when analyzing the TN load of tea plantations in the whole watershed, the changes in TN in sub8 and sub9 must be emphasized. Additionally, urban land accounted for the largest percentage in each subwatershed, with an average TN output of 57.6%. Similar to the TN load results, sub9 had the highest proportional TP load in tea plantations, and the lowest proportion was found in sub1. Because the tea planting area was relatively small in sub1 and sub2, the TP input of tea plantations was also relatively small. However, the tea plantation area in sub9, sub8, sub7, and sub6 was large, and the proportional N and P outputs in these subwatersheds were also large; these conditions were considered in the subsequent evaluation to identify the best reduction management measures.

Determining the loading of TN (the sum of NH_4_-N and NO_3_-N) and TP into rivers under various land-use types is critical to understanding the contributions of different land-use types to N and P inflow loads in a watershed. The SWAT model can be used to evaluate and screen best management practices (BMPs) to prevent agricultural nonpoint-source pollution in a watershed by simulating the relative effects of different agricultural scenarios. In this study, when using the SWAT model to simulate chemical fertilizer reduction scenarios, three types of scenarios were set: nonreduction fertilizer application scenarios (O0), chemical fertilizer reduction scenarios (C1, C2, and C3), and organic fertilizer substitution scenarios (O1, O2, and O3). Details are given in Table 2. The simulation results showed that the N and P loads differed substantially among the different fertilizer reduction scenarios. Under the O0 scenario, the TN load in the whole watershed was 256.6 t/mon, and the TP load was 36.5 t/mon. Regarding the reduced application of pure fertilizer, under the C1 scenario, the TN load was 253.4 t/mon, which was 1.3% lower than that under the O0 scenario, and the TP load was 36.2 t/mon, which was 0.8% lower than that under the O0 scenario. Similarly, under the C2 and C3 scenarios, the TN load decreased by 2.1% and 2.7%, respectively, compared with that under O0, and the TP load was decreased by 1.9% and 3%, respectively. With an increasing degree of reduction, the TN and TP loads of the watershed decreased significantly. Regarding organic fertilizer substitution, under the O1 scenario, the TN and TP loads were 254 t/mon and 36.3 t/mon, respectively, which were 1% and 0.6% lower, respectively, than those under the O0 scenario. Under the O2 and O3 scenarios, compared with that under the O0 scenario, the TN load decreased by 1.5% and 2.1%, respectively, and the TP load decreased by 1.6% and 3%, respectively.

### 3.3. Verification of the Potential Effects of Chemical Fertilizer Reduction on Tea Plantations at the Field Scale

**Figure 4:**
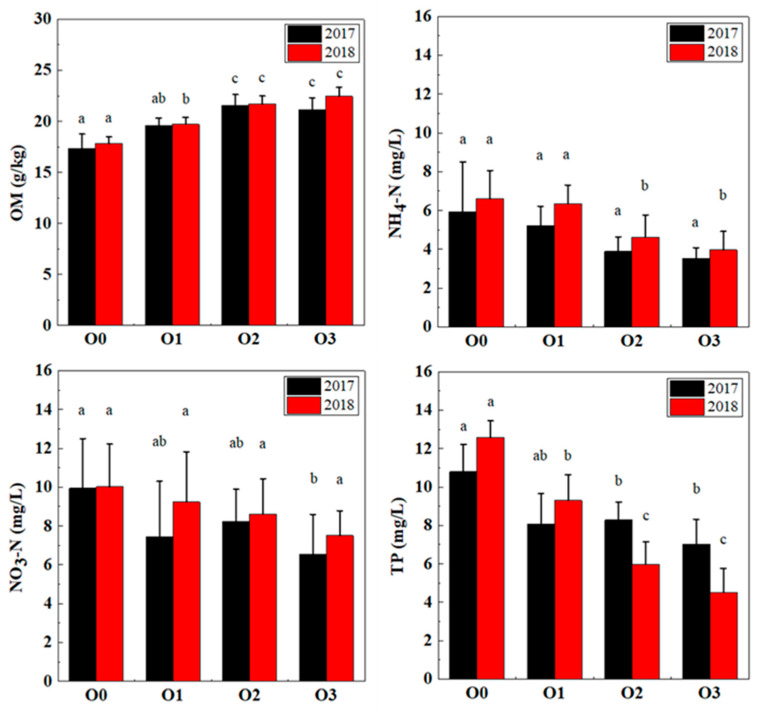
Tea plantation soil OM, runoff water NH_4_-N, NO_3_-N, and TP in the two-year reduction experiment. Note: values followed by different letters are significantly different (*p* ≤ 0.05).

The effects of chemical fertilizer reduction and organic fertilizer substitution on the soil and runoff water of the tea plantation in the field experiment are shown in Figure 4. Before the experiment began, the average SOM content of the tea plantation was 15.9 g/kg. After fertilization, the average SOM content under O0 was 17.4 g/kg in 2017 and 17.8 g/kg in 2018. In 2017, compared with the SOM content under O0, the SOM content of the chemical fertilizer treatments increased by 13% (O1), 24.2% (O2), and 21.6% (O3). In 2018, the SOM content of each treatment increased by 10.7% (O1), 21.7% (O2), and 26.1% (O3). The increase rate of SOM increased with greater organic fertilizer substitution. The increase in SOM was statistically significantly different by year between the O1 treatments, but not in the O2 and O3 treatments. SOM is an essential indicator of soil fertility and improves the effectiveness of soil nutrients, promoting the formation of aggregate structure and enhancing soil buffering [33,34]. The SOM content primarily determines the level of soil fertility. Rapeseed cake organic fertilizer, which was used in this study, provides soils with more organic matter than chemical fertilizers when applied in equal quantities. The reason is that rapeseed cake contains more organic matter than fertilizers themselves, and this organic matter can be slowly decomposed after being introduced to the soil, rather than being dissolved by rainfall and quickly absorbed into runoff, as is the case with fertilizers [35].

In 2017, the average content of NH_4_-N in runoff water from the experimental tea plantation was 0.8 mg/L. After fertilization, the NH_4_-N content of O0 increased correspondingly, with averages of 5.9 mg/L in 2017 and 6.6 mg/L in 2018. Compared with that under O0, the NH_4_-N content in the runoff water of the organic fertilizer substitution treatments decreased by 12.1% (O1), 34.4% (O2), and 40.8% (O3) in 2017 and 3.8% (O1), 29.8% (O2), and 39.9% (O3) in 2018. The NH_4_-N content of runoff water decreased the most in the treatments with organic fertilizer substitution. The NO_3_-N in runoff water of O0 increased from 1.2 mg/L before fertilization to 9.9 mg/L in 2017 and 10 mg/L in 2018 after fertilization. Compared with that under O0, the NO_3_-N content in the runoff water of the organic fertilizer substitution treatments decreased by 25.1% (O1), 17% (O2), and 34% (O3) in 2017 and 7.9% (O1), 14% (O2), and 25.2% (O3) in 2018. A similar trend also occurred in the TP input of runoff water, and the TP input gradually decreased with increases in the substitution proportion of organic fertilizer. The TP input of the organic fertilizer substitution treatment decreased by 27.8% and 47.6% on average, and the reduced amount in 2018 was significantly higher than that in 2017. The largest reduction was observed in the treatment with 80% substitution of organic fertilizer, with decreases of 34.9% and 64.2% in 2017 and 2018, respectively.

**Figure 5:**
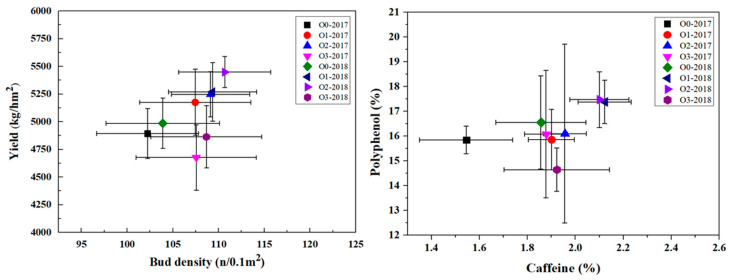
Variation in the tea yield, bud density, and tea polyphenol and caffeine contents in the two-year reduction experiment.

The effects of chemical fertilizer reduction and organic fertilizer substitution reduction on tea yield and quality in tea plantations are shown in Figure 5. Before fertilization, the bud density and tea yields were 100.3 n/m^2^ and 4473.9 kg/m^2^, respectively. After fertilization, the bud density of O0 was 102.2 n/m^2^ in 2017 and 103.9 n/m^2^ in 2018, and the tea yield was 4893.3 kg/m^2^ in 2017 and 4986 kg/m^2^ in 2018. Organic fertilizer substitution significantly increased the bud density, but no significant difference was found between the substitution treatments with different proportions. Compared with that under O0, the bud density of the organic fertilizer substitution treatments increased by 5.1% (O1), 6.7% (O2), and 5.2% (O3) in 2017 and 5.2% (O1), 6.5% (O2), and 4.6% (O3) in 2018. The substitution of organic fertilizer increased the yield of tea in the O1 and O2 treatments. The yield of the O2 treatment increased the most, reaching 7.2% in 2017 and 9.3% in 2018. By contrast, when the substitution of organic fertilizer reached 80%, the yield of tea decreased. Compared with that under O0, the yield under O3 was decreased by 4.4% in 2017 and 2.5% in 2018. Because tea is the most widely used beverage worldwide, the contents of caffeine and polyphenols in tea leaves are two important tea quality indexes. Before fertilization, the average caffeine content and polyphenol content of tea leaves were 1.4% and 14.9%, respectively. After fertilization, the caffeine content of tea leaves in the O0 treatment reached 1.5% and 1.9%, and the tea polyphenol content reached 15.8% and 16.6%; both indicators increased. The caffeine and tea polyphenol contents in the organic fertilizer substitution treatments were slightly higher than those in the O0 treatment, but the differences among treatments were not significant. O2 yielded the largest increase in caffeine content from that under O0 in 2017 (26.6%), while O1 yielded the largest increase in caffeine content in 2018 (14.4%). Regarding tea polyphenols, O2 yielded the largest increases from the levels under O0, increasing by 1.6% in 2017 and 5.6% in 2018. Thus, the best yield and quality of tea were obtained when organic fertilizer was substituted at 50%.

## 4. Discussion

Based on the above analysis, at the provincial scale, the planting conditions and actual amount of chemical fertilizer application in the region must be clarified. The method of spatial analysis is suitable for large areas at the provincial scale; for smaller scales, such as the watershed scale, simulation can be used to achieve a more detailed analysis. Additionally, the simulation method can obtain not only the spatial distribution of chemical fertilizer reduction but also the temporal distribution of tea plantations in the watershed under more complex fertilizer application scenarios. Studies have shown that rainfall is the main driver of agricultural nonpoint source N and P loads, and changes in its total amount and intensity directly affect the mechanism of N and P loss in a watershed [36]. The simulations of NH_4_-N, NO_3_-N, and TP in the present study indicated that the NO_3_-N load was the largest, followed by the NH_4_-N and TP loads. Subwatersheds 1, 2, and 3 had the largest N and P loads in the whole watershed. Because sub1, sub2, and sub3 are downstream from the whole watershed, they collect more pollution than other subwatersheds from upstream, resulting in higher N and P loads than in the upstream subwatersheds. By contrast, sub1, sub2, and sub3 are located in a plain area, in a valley, and are urban areas with concentrated populations. Extensive farmland and concentrated industries are found in these areas, which can be expected to increase N and P loads in these subwatersheds [35]. In particular, sub1, the catchment outlet of the whole watershed, is also the central urban area of the region. Multisource convergence resulted in the N and P loads of sub1 being significantly higher than those of the other subwatersheds. Therefore, in the subsequent simulation of chemical fertilizer reduction measures, the focus should be directed to the characteristics of N and P load changes in sub1, sub2, and sub3.

SWAT model simulation is the most practical and effective method to quantitatively assess N and P loads in tea plantations [37,38]. The simulation results showed that as the reduction in fertilizer application increased, the TN and TP loads in the watershed gradually decreased. The N and P loads of tea plantations exhibit strong spatial heterogeneity, which is closely related to the landforms, climate conditions, soil properties, and fertilizer application management in the watershed. In contrast to the complex N and P input sources of urban land, the N and P inputs from tea plantations are mainly from a single source—chemical fertilizer application; thus, the total amounts of N and P input are much smaller than those of urban land. Additionally, even when the chemical fertilizer application was reduced by 80%, TN decreased by less than 2.7%, while TP decreased by less than 3%; these values represent small reductions for the entire watershed. More importantly, the amount of fertilizer reduction was not proportional to the reduction in the N and P outputs in the final runoff. For example, the fertilizer reduction amount of C3 was 4 times that of C1, but the TN and TP outputs of C3 were only 2.1 times and 3.8 times those of C1, respectively. These findings showed that the effect of chemical fertilizer reduction does not change in proportion to the reduction, with increasing reduction yielding diminishing returns. Additionally, the advantages of pure chemical fertilizer reduction over organic fertilizer substitution were not obvious; with increases in the reduction ratio, the effects of the two methods gradually approached one another. For example, the variation ratio of TP in O1 was 75% of that in C1, that in O2 was 84.2% of that in C2, and those in O3 and C3 were equal. Because the temporal characteristics of chemical fertilizer reduction are different from those of organic fertilizer substitution regarding nutrient loss, the reduction of chemical fertilizer results in a quick short-term effect. Thus, concerning short-term effects, pure chemical fertilizer reduction has better potential than organic fertilizer substitution in reducing TN and TP input in runoff at the watershed scale. At the same time, it must be pointed out that the use of organic fertilizer should not be considered as the more the better, because it may also be a potential source of environmental pollution, such as eutrophication of water bodies, so appropriate measures should be taken to use organic fertilizer [39,40]. Furthermore, the simulation showed that a low-proportional reduction or organic fertilizer substitution agrees more with actual production requirements than a large reduction (e.g., 80%) in pure chemical fertilizer use. In this way, the soil fertility of tea plantations can be effectively supplemented to promote the growth of tea trees, and the N and P outputs can be reduced to achieve optimal production conditions. Therefore, regarding long-term effects, organic fertilizer substitution is the best strategy and the measure with a higher potential for chemical fertilizer reduction [41]. Additionally, determining the differences in the proportions of land-use patterns within a watershed is critical to analyzing the potential for chemical fertilizer reduction at the watershed scale.

Heavy rains over a short period usually lead to the rapid leaching of N and P from fertilizers into runoff water. This phenomenon is common in Zhejiang Province, which is rich in precipitation [42]. Studies have also shown that most of the chemical fertilizers applied to tea plantations will be incorporated into runoff water when precipitation occurs, and only a small part can be stored in the soil and used by tea trees. This phenomenon also explains why excessive fertilization easily causes N and P nonpoint-source pollution [43]. Organic fertilizer substitution not only increases the SOM content but also increases the soil water retention rate. Organic fertilizer application also retains more nutrients, such as N and P, in the soil [44]. On the one hand, as an ammonia-loving plant, tea trees can absorb large amounts of NH_4_-N, resulting in a relatively small amount of NH_4_-N flowing into runoff water compared with the amount of NO_3_-N. On the other hand, NH_4_-N can be easily absorbed by soil particles and SOM; under aerobic soil conditions, the nitrification activity of tea plantation soils is very high. When organic fertilizer is applied, the number of nitrifying bacteria will increase, resulting in NH_4_-N changing rapidly to NO_3_-N [45]. Furthermore, the nitrification capability of organic fertilizer is lower than that of chemical fertilizer [46]. Therefore, appropriate organic fertilizer substitution (such as 50% organic fertilizer substitution) is an effective strategy to reduce fertilizer application in tea plantations and the probability of N and P pollution.

Tea has an attractive aroma, good taste, and health-promoting effects, and these benefits lead to its consumption by more than two-thirds of the world’s population [47,48]. The caffeine content of tea can directly affect its taste, and caffeine has a refreshing effect, while tea polyphenols are the most biologically active group of tea components and have important applications in the food industry for daily use [49]. Therefore, when considering the effects of chemical fertilizer reduction, the influence on tea quality (particularly caffeine and polyphenols) should be considered. If the quality of tea is seriously affected, independent of the actual production demand, the significance of fertilizer reduction will be substantially reduced. Organic fertilizer substitution can effectively influence tea yield and improve the quality of green tea by boosting the caffeine and polyphenol contents of tea. Although the yield will not continue to increase when the substitution rate exceeds a certain proportion, such as 80%, there may be a trend leading to a decrease in tea yield. This conclusion is consistent with the simulation results of the SWAT model for tea plantations at the watershed scale. In terms of long-term effects, 50% organic fertilizer substitution is the best strategy because it can effectively balance the two aspects of tea growth and environmental protection and is the measure with a higher potential for chemical fertilizer reduction.

## 5. Conclusions

A reduction in chemical fertilizer use in tea plantations in Zhejiang Province is needed, and the analysis of reduction potential under different spatial scales is critical to formulating targeted reduction strategies. The province-level analysis showed that 51.7% of the area had an average total fertilization intensity greater than 350 t/hm^2^ with a high reduction potential. The accurate analysis of planting suitability and fertilization status is crucial to determine the potential for application reduction at the provincial scale. The watershed analysis revealed that both the TN load and TP load in the watershed were reduced under the chemical fertilizer reduction scenarios and organic fertilizer substitution scenarios. Evaluating the differences in land-use patterns is key to analyzing the potential for chemical fertilizer reduction at the watershed scale. Pure chemical fertilizer reduction has better potential in reducing TN and TP inputs in runoff concerning short-term effects, whereas organic fertilizer substitution is the best strategy concerning long-term effects. This conclusion was also reached in the quantitative tea plantation field experiments. An appropriate percentage (such as 50%) of organic fertilizer substitution is the best strategy to balance tea yield, tea quality, and environmental protection and is the measure with the higher potential for chemical fertilizer reduction. This study provides a useful method to investigate strategies to reduce chemical fertilization in tea-growing areas at the province, watershed, and field levels.

## Figures and Tables

**Table 1:** Details of the application of fertilizer types and doses.

Fertilizer Treatment	Type and Dose of Fertilizer (kg/hm^2^)				
	Compound Fertilizer	Urea	Calcium Superphosphate	Potassium Sulfate	Rapeseed Cake
100% chemical fertilizer (0% organic fertilizer substitution) (O0)	600	782	500	350	-
20% organic fertilizer substitution (O1)	-	761	1049	480	1904
50% organic fertilizer substitution (O2)	-	478	995	450	3928
80% organic fertilizer substitution (O3)	-	191	870	421	6207

Note: The annual application rate of each treatment was 450 kg/hm^2^ in terms of pure nitrogen, while the ratio of N:P_2_O_5_:K_2_O was 3:1:2.

**Table 2:** Simulation results of TN, TP, and the variable rate under three types of fertilizer reduction scenarios.

Fertilizer Reduction Scenario	Description	Code	TN (t/mon)	Variable Rate (%)	TP (t/mon)	Variable Rate (%)
Nonreduction fertilizer application	100% application	O0	256.6	-	36.5	-
Chemical fertilizer reduction	Reduction of 20%	C1	253.4	−1.3	36.2	−0.8
	Reduction of 50%	C2	251.3	−2.1	35.8	−1.9
	Reduction of 80%	C3	249.6	−2.7	35.4	−3.0
Organic fertilizer substitution	Substitution of 20%	O1	254.0	−1.0	36.3	−0.6
	Substitution of 50%	O2	252.8	−1.5	35.9	−1.6
	Substitution of 80%	O3	251.1	−2.1	35.4	−3.0

## Data Availability

The associated dataset of the study is available upon request to the corresponding author.

## Supplementary Materials

The following are available online at https://www.mdpi.com/article/10.3390/ijerph19095243/s1. Table S1: Average planting area of crops and amount of fertilizer applied in all regions of Zhejiang Province from 2008 to 2017; Table S2: Specific rating criteria for climate factors, soil factors, and topographic factors; Table S3: Rating of chemical fertilizer reduction potential in tea plantations of Zhejiang Province; Figure S1: Distribution of monitoring points, meteorological stations, and land-use types in the studied basin; Figure S2: Runoff, NH_4_-N, NO_3_-N, and TP load calibration and validation of the SWAT model; Figure S3: Schematic diagram of the tea garden field experiment layout; Figure S4a: Ratios of the total nitrogen load under different land-use patterns in each subwatershed; Figure S4b: Ratios of the total phosphorus load under different land-use patterns in each subwatershed.
