# Supplementary material for: Potential to Reduce Chemical Fertilizer Application in Tea Plantations at Various Spatial Scales
Source: Int J Environ Res Public Health. 2022 Apr 26;19(9):5243. doi: 10.3390/ijerph19095243 (PMC9103282; doi:10.3390/ijerph19095243)
Supplement: Supplementary file 1 [file ijerph-19-05243-s001.zip › ijerph-1651285-supplementary.pdf]

International Journal of
Environmental Research and Public Health

MDPI

Supplementary Materials

Table S1. Average planting area of crops and amount of fertilizer applied in all regions of Zhejiang Province from 2008 to 2017.

Cities	Crop sown area (thousand hectares)	Nitrogen fertilizer application (10,000 tons)	Phosphate fertilizer application (10,000 tons)	Potassium fertilizer application (10,000 tons)	Compound fertilizer application (10,000 tons)	Total fertilizer application (10,000 tons)
Hangzhou	345.74	5.13	1.19	0.8	3.43	10.55
Ningbo	302.04	5.03	1.53	0.85	3.57	10.98
Jiaxing	326.27	7.74	1.22	0.54	0.87	10.37
Huzhou	204.37	3.08	0.52	0.29	1.16	5.05
Shaoxing	302.14	7.06	0.84	0.67	2.02	10.59
Zhoushan	21.09	0.25	0.06	0.02	0.17	0.5
Wenzhou	236.52	4.93	1.38	0.86	1.28	8.45
Jinhua	250	4.9	1.61	1.12	3.42	11.05
Yiwu	27.27	0.53	0.14	0.1	0.51	1.28
Quzhou	214.65	3.91	0.88	0.85	1.73	7.37
Taizhou	239.8	4.71	0.92	0.51	2.84	8.98
Lishui	168.73	2.63	0.94	0.62	1.9	6.09
All provinces	2613.55	49.37	11.08	7.13	22.38	89.96

Table S2. Specific rating criteria for climate factors, soil factors and topographic factors.

Climate factor	Annual average illumination	Annual average precipitation	Annual average temperature	Annual average relative humidity	Factor rating
	34-37%	1000-1300 mm	18-19°C	75.5-77%	1
	37-40%	1300-1600 mm	17-18°C	77-78%	2
	40-45%	1600-2000 mm	16-17°C	78-79.5%	3
Soil factor	Land use type	Soil type	Soil texture	Soil erosion degree	Factor rating
	e.g., woodland and shrubland	e.g., yellow brown soil and red soil	Percentage of loam, 0-20%	Slight erosion	1
	e.g., paddy field and dry land	e.g., lime soil and coarse bone soil	Percentage of loam, 21-40%	Moderate erosion	2
	e.g., high cover grass and tidal flats	e.g., mountain meadow soil and coastal salt soil	Percentage of loam, >40%	Strength erosion	3
Topographic factor	Elevation	Slope	Aspect		Factor rating
	0-500 m	0-20°	Southeast, south, southwest		1
	500-1500 m	20-40°	East and west		2
	>1500 m	>40°	Northeast, northwest and north		3

Table S3. Rating of chemical fertilizer reduction potential in tea plantations of Zhejiang Province.

Fertilizer reduction suitability	Tea planting suitability	Rating result	Area percentage
Most suitable reduction area	Most suitable tea planting area	Class 1	16.6
	Subsuitable tea planting area	Class 2	21.9
	Generally suitable tea planting area	Class 3	14.5
Subsuitable reduction area	Most suitable tea planting area	Class 4	11
	Subsuitable tea planting area	Class 5	16.5
	Generally suitable tea planting area	Class 6	13.7
Generally suitable reduction area	Most suitable tea planting area	Class 7	2.1
	Subsuitable tea planting area	Class 8	2.1
	Generally suitable tea planting area	Class 9	1.6

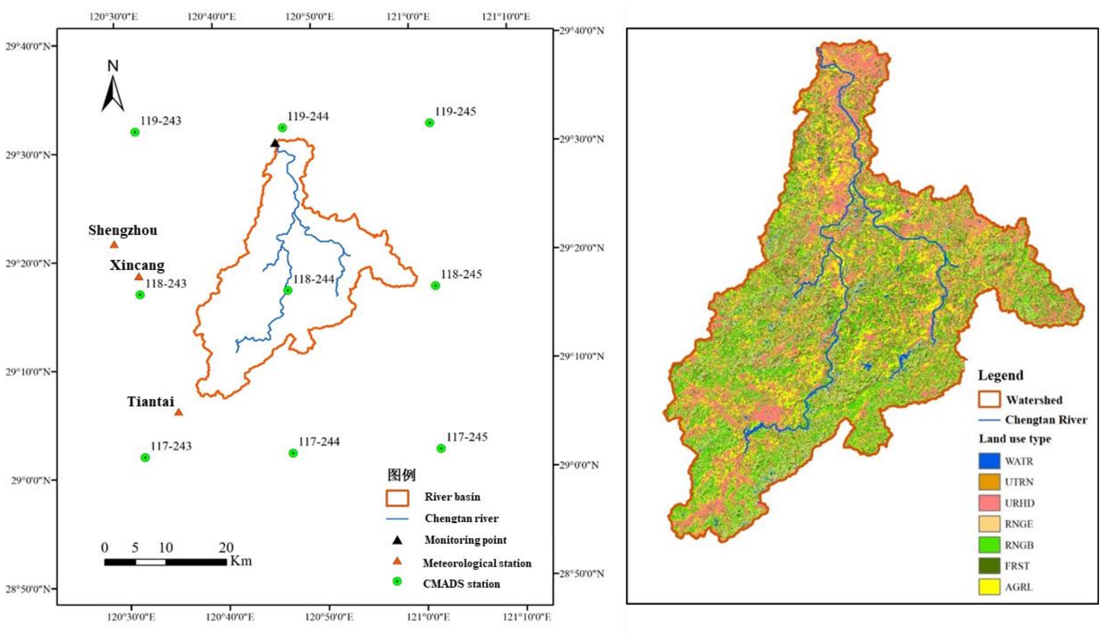

Figure S1. Distribution of monitoring points, meteorological stations and land use types in the studied basin.

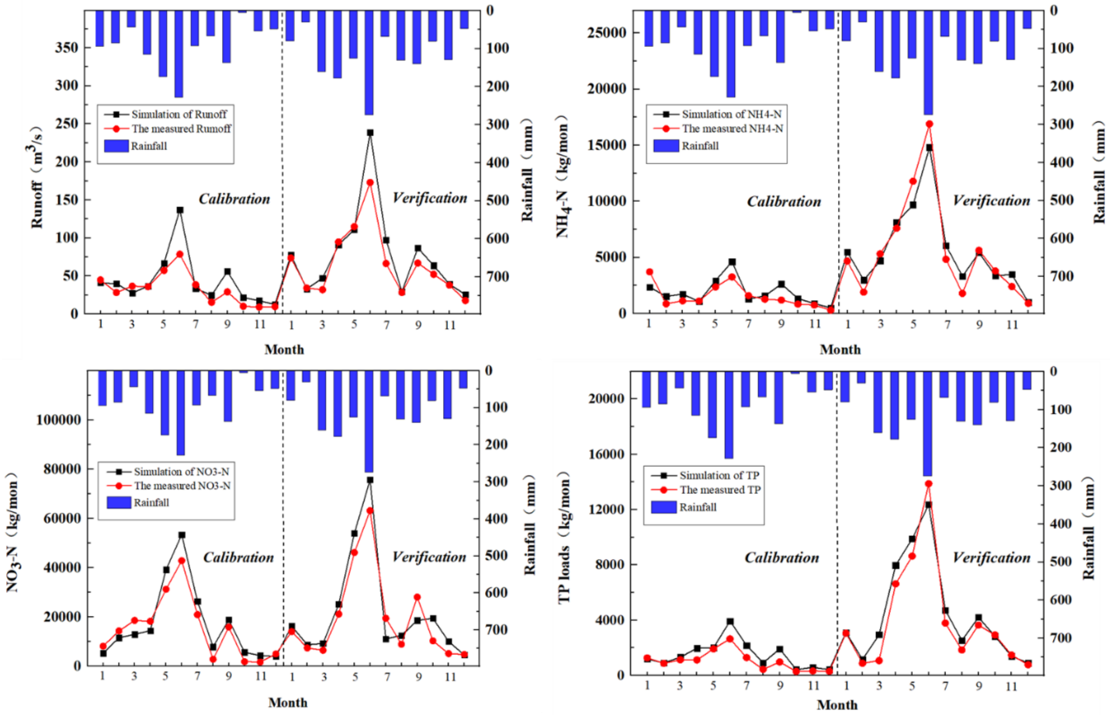

Figure S2. Runoff, NH_4-N, NO_3-N and TP load calibration and validation of the SWAT model.

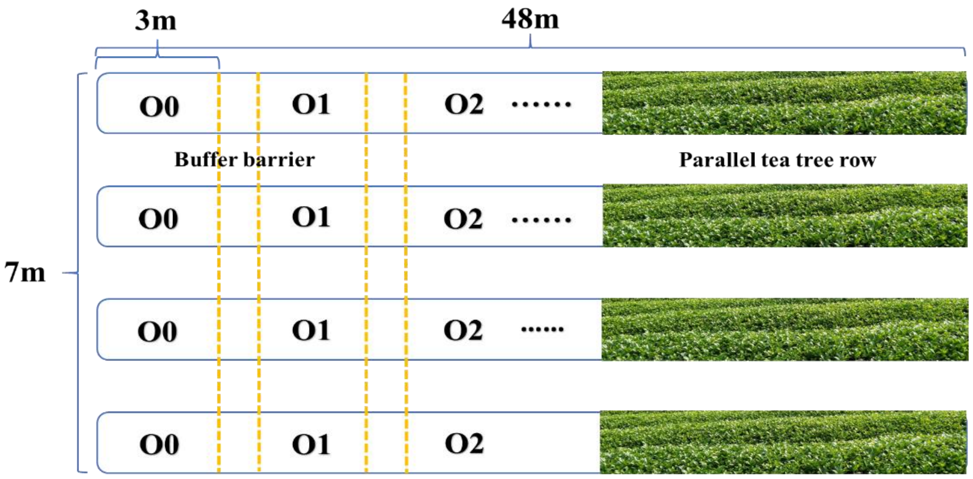

Figure S3. Schematic diagram of the tea garden field experiment layout.

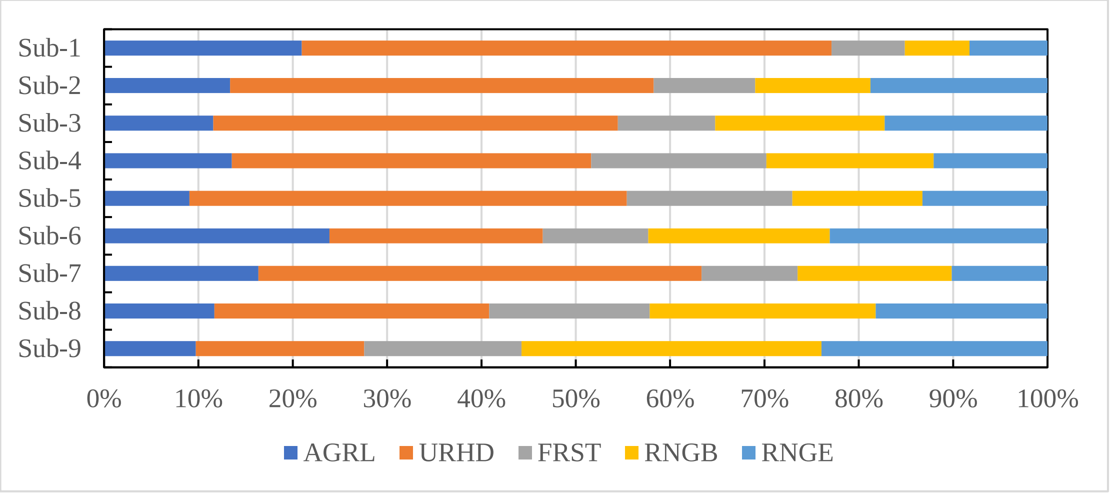

(**a**)

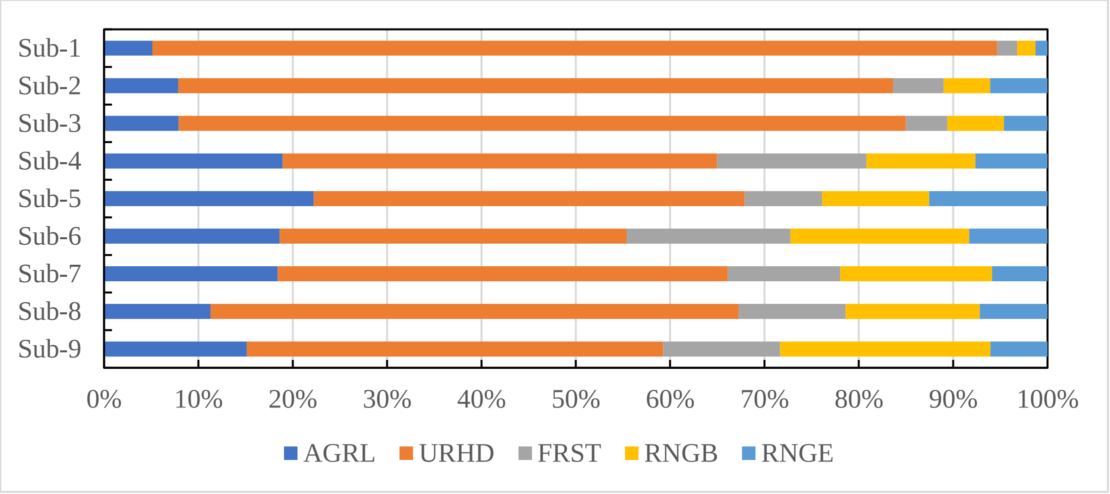

(**b**)

Figure S4. (**a**) Ratios of the total nitrogen load under different land use patterns in each subwatershed; (**b**) Ratios of the total phosphorus load under different land use patterns in each subwatershed.Note: arable land (AGRL), urban land (URHD), barren grassland (RNGE), tea plantation, land (RNGB) and forestland (FRST)
